# The positive allosteric modulator GNE-9278 increases gating, conductance, and Ca^2+^ permeability for GluN2D-containing NMDA receptors

**DOI:** 10.1038/s41386-025-02308-8

**Published:** 2026-01-07

**Authors:** Jamie A. Abbott, Mae G. Weaver, Beiying Liu, Samantha R. Schwarz, Gabriela K. Popescu

**Affiliations:** Department of Biochemistry, Jacobs School of Medicine and Biomedical Sciences, University at Buffalo, Buffalo, NY USA

**Keywords:** Pharmacology, Ion channels in the nervous system

## Abstract

Positive allosteric modulators (PAMs) of NMDA receptors hold promise to alleviate functional deficits associated with neuropsychiatric disorders. GNE-9278 is a synthetic PAM that binds to the obligatory GluN1 subunit of NMDA receptors, yet it displays substantial subtype-specific efficacies. To examine the underlying mechanism by which GNE-9278 potentiates NMDA receptor responses, we examined electrical activity from endogenous and recombinant NMDA receptors and measured changes in kinetics, unitary conductance, and Ca^2+^ permeability for GluN2A- and GluN2D-containing receptors. We found that GNE-9278 stabilized the open states of both receptor types and increased their open state occupancies at equilibrium. This change was small for GluN2A receptors, whose open probability (Po), remained around 0.5, and was substantial for GluN2D, which increased four-fold to 0.24. Moreover, specifically when acting on GluN2D receptors, GNE-9278 increased the channel unitary conductance (γ_Na_), from 43 pS to 56 pS, and their Ca^2+^ permeability (P_Ca_/P_Na_), from 1.4 to 2.9. These results show large and complex effects of GNE-9278 on GluN2D receptors, which together boost these receptors’ excitatory signal and impact on synaptic plasticity and cellular fate. This new information should guide the use of drugs acting at the GNE-9278-binding site for research and therapeutic purposes.

## Introduction

NMDA receptors are glutamate-gated excitatory ion channels with large and dynamic Ca^2+^ permeability [1–4**]**. They mediate fast excitatory transmission at central synapses, are essential for synapse formation and plasticity, and regulate cellular fates during development and across the lifespan. Several neuropsychiatric disorders have been linked to NMDA receptor dysfunctions and small molecules that modulate NMDA receptor currents by direct and reversible binding at allosteric sites are currently investigated as potential therapies [5, 6]. Specifically, NMDA receptor positive allosteric modulators (PAM) may prove useful in alleviating symptoms resulting from glutamatergic hypofunction, including neurodevelopmental conditions such as schizophrenia, epileptic encephalopathies, autistic spectrum disorders, and cognitive delays [7].

**Fig. 1:**
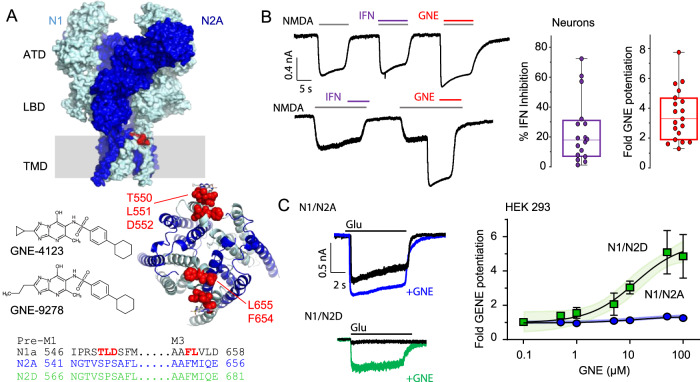
GNE-9278 potentiates NMDA receptor responses. **A** *Top*, atomic arrangement of an NMDA receptor (PDB: 9C7C) illustrates GluN1 (N1, light blue) and GluN2A (N2A, dark blue) subunits, and highlights residues interacting with GNE-4123 (red). *Below*, chemical structures of two related PAMs, and top view of the receptor’s trans-membrane domain. *Bottom*, sequence alignment of GluN subunits highlights PAM-interacting residues (red). **B** *Left*, representative whole-cell current traces elicited from primary neurons (DIV, 12-19) with NMDA (0.1 mM) and the indicated drug (IFN, 10 µM; GNE-9278, 50 µM), as indicated. *Right*, box plots illustrate pooled results for drug-induced change in the steady-state NMDA response. **C** Representative whole-cell current traces elicited with Glu (1 mM) from recombinant GluN1/GluN2A (N1/N2A, top) and GluN1/GluN2D (N1/N2D, bottom) receptors (gray) and superimposed responses obtained after adding GNE-9278 (50 µM) (black). *Right, s*ummary of results (symbols) and dose-response relationships fitted to the data (lines).

NMDA receptors are heterotetramers composed of two obligatory GluN1 subunits and two GluN2 subunits, of which four distinct isoforms (A-D) have regulated expression and impart distinctive biophysical properties (Fig. 1A) [1, 8, 9]. GNE-9278 and GNE-4123 are related synthetic triazolo-pyrimidine sulfonamide-containing molecules and pan-NMDA receptor PAMs, which bind to residues on the GluN1 subunit, close to the receptor’s transmembrane helices (Fig. 1A) [10, 11]. Importantly for its prospective therapeutic value, the efficacy with which GNE-9278 potentiates NMDA receptor currents depends on the receptor’s molecular composition, being substantially more effective at enhancing responses from GluN2C- and GluN2D-containing receptors (15-fold), rather than those from GluN2A- or GluN2B-containing receptors (5-fold) [10]. Given the widespread expression of NMDA receptors in brain and spinal cord, modulators with subunit-specific potencies or efficacies have the advantage of more targeted effects. As such, there is substantial interest in understanding their mechanism of action.

Previous studies showed that GNE-9278 increases the receptors’ peak currents and slows their deactivation kinetics [10]. Moreover, GNE-9278 interferes with the receptor’s sensitivity to agonists and to allosteric modulators [10, 12], and stabilizes open conformations of GluN2A receptors [11]. Here, we build on these findings and examine whole-cell and single-molecule currents elicited with suprasaturating concentrations of agonists and in nominal absence of blockers or inhibitors, to delineate the intrinsic effects of GNE-9278 on the biophysical properties of GluN2A and GluN2D receptors.

## Materials and methods

### Chemicals and reagents

Reagents used for electrophysiological recordings were purchased from Millipore-Sigma and were of >99.0% purity. GNE-9278 (Catalog number 6369; batch numbers 1 A/245695 and 1B/288164) and 1,2-bis(o-aminophenoxy) ethane-N,N,N′,N′-tetraacetic acid (BAPTA) (CAS: 126150-97-8) were purchased from (Tocris Bioscience). Tissue culture reagents were purchased from Corning Life Sciences. HEK293 cells and plasmids were utilized under the approved Safety Protocol ID: 030BIO00000155, from the University at Buffalo Institutional Biosafety Committee.

### Cells and plasmids

All reagents for neuronal cell culture were purchased from Gibco. Round 14 mm coverslips for culturing primary cortical neurons were nitric acid washed, autoclaved and coated with poly-D-lysine. Rat cerebral cortex neurons (Gibco, A36512, lot 2537941) were plated at a density of 125,000 and 75,000 and cultured at 37 °C and 5% CO_2_ in a Neurobasal medium with B-27, N2 and glutamax growth supplements. Electrophysiology experiments on neurons were performed on days 12–19 of cultivation. Cortical neurons were bathed in solution containing (in mM) 140 NaCl, 5 KCl, 2 CaCl_2_, 2 MgCl_2_, 10 HEPES, and 10 glucose, pH adjusted with NaOH to 7.4 during electrophysiology experiments. Extracellular solution used during macroscopic recordings contained (in mM); 150 NaCl, 2.5 KCl, 0.5 CaCl_2_, 10 HEPBS, 0.1 EDTA, and 0.1 glycine and pH adjusted with NaOH to 8.0, while intracellular solution contained (in mM): 135 K-gluconate, 7.5 KCl, 10 HEPES, 10 mM phosphocreatine, 2 ATP, 0.3 GTP and pH 7.3. Application of NMDA at 100 μM was used to elicit stimulate endogenous NMDA receptors.

HEK-293 cells (ATCC CRL-1573) were maintained in Dulbecco’s Modified Eagle Medium (DMEM) supplemented with 10% fetal bovine serum and 1% pen-strep, at 37 °C, in a 5% CO_2_ atmosphere and were passaged when reaching 80–90% confluence. Plasmids used were rat GluN1-1a (GenBank: U08261.1) and rat GluN2A (GenBank: AF001423.1) in pcDNA3(+), rat GluN2D (GenBank: L31611.1) in pCI-Neo (with P94R and A305R variants as previously reported in reference NM_022797.2) and gWIZ-GFP plasmid (Genlantis, a division of Gene Therapy Systems, Inc.). Complete sequences were confirmed by Sanger sequencing. Cells were transiently transfected with polyethylenimine linear, MW 25,000 (Polysciences, Inc.) in a 5:1 ratio (PEI: DNA, by mass). Following transfection, cells were incubated 24–48 h. in growth medium supplemented with 2 mM Mg^2+^ to prevent NMDA receptor-mediated cell death. Before each experiment, cells were washed, covered with Dulbecco’s Phosphate Buffered Saline (DPBS) and placed on the stage of an inverted microscope. Individual cells were selected visually for patch-clamp recordings, based on the fluorescence intensity.

### Unitary current recordings and analyses

**Fig. 2:**
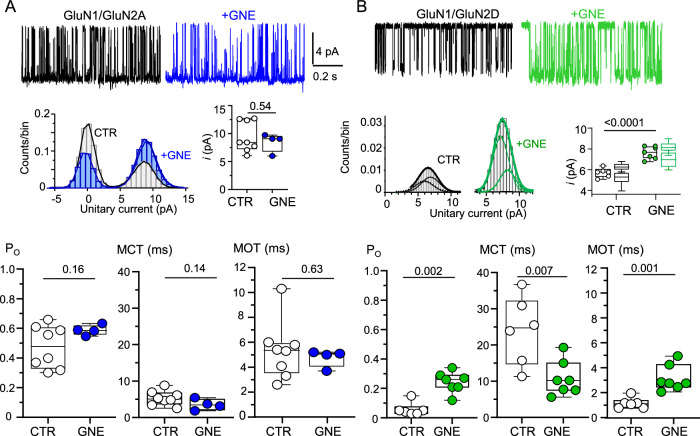
Effect of GNE-9278 on single-molecule currents. *Top*, traces illustrate portions of continuous inward sodium currents recorded from individual GluN1/GluN2A (**A**) and GluN1/GluN2D (**B**) receptors expressed in HEK293 cell, without and with GNE-9278 ( + GNE, 50 µM, color). *Below*, histograms of unitary current amplitudes from one recording overlaid with probability distribution functions (lines), and box plots of summary results (*right*). *Bottom*, summary of results for open probabilities (Po), mean open (MOT) and mean closed (MCT) times in the absence (open circles) and presence (filled circles) of GNE-9278. All *p* values were calculated with Student’s t-tests.

Unitary sodium currents were recorded from individual receptors with the cell-attached patch-clamp technique as described previously [13]. Briefly, polished glass electrodes were filled with extracellular solutions containing (in mM): 150 NaCl, 2.5 KCl, 1 EDTA, 10 HEPBS adjusted to pH 8.0 (NaOH) and the agonists glycine (0.1 mM) and glutamate (1 mM), as optimized previously for adequate resolution of NMDA receptor gating events [14]. Inward currents were recorded after applying +100 mV through the recording pipette with an estimated final membrane-patch potential of ~ −120 mV. Currents were amplified and low-pass filtered at 10 kHz (Axopatch200B; 4-pole Bessel), sampled at 40 kHz (PCI-6229, M Series card, National Instruments, Austin, TX), and written into digital files with QuB acquisition software. Recordings were preprocessed off-line to correct baseline drifts and spurious electro-mechanical noise. Idealization was done with the segmental-k-mean (SKM) method in QuB with a 0.025 ms deadtime and no digital filtering. For analyses, we retained only recordings devoid of double openings and of sufficient duration to ascertain the presence of a single channel as described previously [15]. Unitary current amplitude (*i*), open probability (Po), mean open (MOT), and mean closed (MCT) times were estimated for each recording using all the detected events in each digital file (2.8 ×105 – 1.2 ×106 events) (Fig. 2, Table S1).

Kinetic modeling was done using the maximum-interval likelihood (MIL) method implemented in QuB software by fitting iteratively to idealized data user-defined state models with increasing number of closed and open states, after imposing a 0.075 ms deadtime as described in detail previously [16]. The final model was selected by maximizing the fit between the probability density function (pdf) calculated with the model and the actual distribution of the data, with the optimal number of states determined according to the Akaike criterion, which requires an increase of 20 LL units per added state. The arrangement of states within the model was imposed a priori based on reported criteria and validation for NMDA receptors [17**–**19].

Unitary calcium currents were recorded as described in detail previously [20]. Briefly, extracellular solutions contained (in mM): 75 CaCl_2_, 5 KCl, 1 EDTA, 0.1 glycine, 1 glutamate and HEPBS (pH 8). Cells were bathed in a KCl-based solution containing (in mM), 142 KCl, 1.8 CaCl_2_, 1.7 MgCl_2_ and 10 HEPES set to pH 7.4 to collapse the cellular resting membrane potential. We determined I-V relationships by measuring unitary current amplitudes in cell-attached preparations at four discrete membrane potentials (−100, −80, −60 and −40), where Ca^2+^ was the dominant permeating ion. We then quantified unitary conductance as the slope of a linear regression fitted to the i-V measurements.

### Macroscopic current recordings and analyses

We recorded whole-cell currents with the patch-clamp technique as described in detail previously [21]. Briefly, glass electrodes were filled with intracellular solutions containing (in mM): 135 CsCl, 10 HEPES, and 10 BAPTA, adjusted to pH 7.2 (CsOH). After reaching the whole-cell configuration, the voltage was clamped at −70 mV and cells were perfused with extracellular solutions using a pressurized perfusion system with exchange time of 0.3–0.5 s (BPS-8, ALA Scientific Instruments). Extracellular solutions contained (in mM): 150 NaCl, 2.5 KCl, 0.5 CaCl_2_, 0.01 mM EDTA and 10 HEPBS buffer, adjusted to pH 8 (NaOH), and co-agonists glutamate (1 mM) and glycine (0.1 mM). Macroscopic currents were amplified and low-pass filtered at 2 kHz (Axopatch200B; 4-pole Bessel), sampled at 5 kHz (Digidata, 1322 A), and written into digital files with pClamp 10.5 acquisition software (Molecular Devices). To observe the effects of GNE-9278, we co-applied increasing concentrations of GNE-9278 together with the agonists and measured peak (Ipk) and steady-state (Iss) current amplitudes (Fig. 1C).

Macroscopic current traces were analyzed using Clampfit (Molecular Devices). Dose-response curves were constructed by measuring current amplitudes before (I_CTR_), and during (I_GNE_) drug application, and plotting values for fold-increase in current amplitudes for each concentration of GNE-9278 tested. The magnitude of the effect was determined as follows:

1 $$\frac{{I}_{{GNE}}}{{I}_{{CTR}}}={MinA}+\,\frac{\left({MaxA}-{MinA}\right)\,}{{1+\left(\frac{{EC}50}{\left[{GNE}\right]}\right)}^{{HillSlope}}}$$

where maximum asymptote is represented by MaxA, and minimum asymptote is represented by MinA. Dose-response curve was assumed to have a standard slope (Hill coefficient of 1.0). All parameters were free to vary during fitting.

### Calcium permeability measurements

We measured relative permeability (P_Ca_/P_Na_) following the biionic method as described previously [2, 22]. Briefly, after cellular break, we held cells at -100 mV to establish the zero-current baseline and then adjusted the membrane potential to +60 mV over a 3 s period recording the non-specific current passing during a voltage-ramp. NMDA receptor currents were elicited with 7 s applications of agonists (1 mM Glu, 0.1 Gly) and the voltage ramp was applied during the steady-state phase of the current response. The protocol was done with extracellular solutions containing either 150 mM NaCl or 75 mM CaCl_2_, with and without GNE-9278, as indicated, to measure the effects of GNE-9278 on the Na^+^ and Ca^2+^ reversal potentials (E_rev_). Relative Ca^2+^ permeability P_Ca_/P_Na_ was calculated with the equation below.

2 $$\frac{{P}_{{Ca}}}{{P}_{{Na}}}=\,\frac{ \left[{Na}\right]({e}^{\frac{\varDelta {E}_{{rev}}}{\alpha }})\,({e}^{\frac{{E}_{{Ca}}}{\alpha }}\,+\,1)}{4\,\left[{Ca}\right]}$$

[Na] and [Ca] are extracellular concentrations of Na^+^ and Ca^2+^ respectively, *α* = RT/F ( ~ 25.4 mV) and E_Ca_ is the reversal potential observed in Ca^2+^ conditions. For drug-free conditions, ΔE_rev_ was measured relative to E_Na_ in the absence of GNE-9278. For drug-containing conditions, ΔE_rev_ was measured relative to E_Na_ in the presence of GNE-9278. In each case, ΔE_rev_ was calculated by accounting for the estimated liquid junction potential, which was estimated at 15.1 mV with LJPCalc (https://swharden.com/LJPcalc/). To interconvert P_Ca_/P_Na_ and Pf, we used the equation below as described previously [23, 24]:

3 $$\frac{{P}_{{Ca}}}{{P}_{{Na}}}={Pf}( \% )\frac{1+\left[{Na}\right]({e}^{\frac{V}{\alpha }}\,+1)({e}^{\frac{V}{\alpha }})}{40\,\left[{Ca}\right]}$$

All statistical analyses were done using R statistical software (v. 4.2.0) or GraphPad Prism (v. 10.5.0). Summary results are given as means with standard deviations (SD) or with 95% confidence interval (CI), as indicated. Statistical significance of differences was determined with a Student’s t-test, with the threshold set to *α* = 0.05.

## Results

### GNE-9278 potentiated macroscopic NMDA receptor currents

We reported recently that when examined in the absence of extracellular inhibitors, such as H^+^ (pH 8) and divalent cations, such as Ca^2+^, Zn^2+^, and Mg^2+^ (EDTA), GNE-9278 had only a marginal effect on the open probability (Po) of receptors containing GluN2A variants [11]. This observation suggests that the main effect of GNE-9278 is to prevent tonic inhibition by ambient ions acting through extracellular domains [10, 25]. If this is true for all NMDA receptors, GNE-9278 should have marginal effect on endogenous NMDA receptor currents when observed in the absence of inhibitory extracellular ions. To test this hypothesis, we recorded whole-cell currents elicited with NMDA (0.1 mm) from rat cortical neurons, maintained in culture for 12 to 19 days (Fig. 1B). We found that relative to control currents, GNE-9278 (50 µM) consistently increased NMDA-elicited currents ( ~ 2 fold), regardless of whether the activity of GluN2B receptors was inhibited by pre- or co-application of the GluN2B-selctive inhibitor ifenprodil (IFN, 10 µM). The effects of both IFN and GNE-9278 varied widely from cell to cell, with 0 to 80% inhibition for IFN and 1.5 to 8-fold potentiation for GNE-9278, suggesting variable expression of NMDA receptor levels and composition in our neuronal cultures. We did not detect any correlation between the effect of IFN and that of GNE-9278, and therefore we pooled data in Fig. 1B.

Based on these results, and on previous reports that GNE-9278 has much higher efficacy for GluN2D-containing receptors (15-fold) [10], we selected to examine and compare the effects of GNE-9278 on GluN1/GluN2A and on GluN1/GluN2D receptors. We constructed dose-response relationships for receptors expressed in HEK293 cells stimulated with suprasaturating agonist concentrations (1 mM Glu, 0.1 mM Gly), in the absence of allosteric inhibition by protons (pH 8) or Zn^2+^ (0.1 mM EDTA), or block by divalent cations (0.25 mM Ca^2+^, 0 Mg) [14]. For GluN2A receptors, the half-maximal effective concentration (EC50) of GNE-9278 was 13 µM (95% CI 4 – 43 µM) with 1.3-fold maximum increase in whole-cell steady-state current. For Glu2D receptors, the EC50 was not different, 13 µM (95% CI 6–32 µM), but the maximal potentiation was 5-fold (Fig. 1C). While much larger than the effect observed at GluN2A receptors, the increase in current was 3-fold smaller than that reported for recombinant GluN2D receptors in physiological concentrations of protons (pH 7.3), suggesting that a substantial portion of the effect of GNE-9278 reflects relief from tonic inhibition by ligands acting at the N-terminal domain [10, 25]. As expected for a PAM, we observed that the effect of GNE-9278 was fully reversible at both receptor types (Fig. S1).

### GNE-9278 increased GluN1/GluN2D open probability and conductance

Next, we aimed to compare the effects of GNE-9278 on the activity of individual GluN2A and GluN2D receptors. We recorded inward sodium currents from cell-attached patches containing only one active receptor (see Methods) with pipettes containing Glu (1 mM), Gly (0.1 mM), and GNE-9278 (50 µM) and compared these with currents recorded in the absence of GNE-9278 (control, CTR) (Fig. 2A, B). For GluN2A receptors, GNE-9278 had no statistically significant effect on the unitary current amplitude (*i*), channel open probability (Po), or mean open time (MOT). Therefore, although GNE-9278 produced a small (1.3-fold) increase in whole-cell currents (Fig. 1A), we were unable to observe changes in microscopic conductance or kinetic parameters for this receptor type. This result was likely due to the modest overall effect of GNE-9278 on GluN2A receptor kinetics and the well-documented complexity of NMDA receptor gating [9].

For GluN1/GluN2D receptors, we observed that channels opened to two amplitude levels: a sub-level of 5.2 ± 0.7 pA with a relative occupancy of (69%) and a main level of 6.3 ± 0.4 pA with a relative occupancy of (31%), corresponding to 36 ± 5 pS and 43 ± 3 pS conductance (Fig. 2B, Table 1), as reported previously [26]. Notably, in the presence of GNE-9278, the unitary current amplitude was ~1.3-fold larger for both open levels: 7.0 ± 0.7 (67%), and 8.2 ± 0.4 pA (33%), respectively, corresponding to unitary conductances of 48 ± 5 pS and 56 ± 3 pS. In addition, the Po was ~4-fold larger, increasing from 0.06 ± 0.05 to 0.24 ± 0.07. This increase in activity reflected a combination of 3-fold longer openings, with MOT increasing from 1.1 ± 0.5 ms to 3.2 ± 1.0 ms, and 2-fold shorter closures, with MCT decreasing from 24 ± 9 ms to 11 ± 5 ms (Fig. 2B, Table S1).

**Table 1:** Summary of GNE-9278 effects on GluN2D Na^+^ and Ca^2+^ unitary conductance.

[GNE]	γ_Na_ (pS)	Sub	p-value	Sub	γ_Ca_ (pS)	Sub	p-value	Sub
(µM)	Main		Main		Main		Main	
-	43.1 ± 2.6	36.0 ± 4.6			12.3 ± 2.2	6.8 ± 2.0		
50	56.4 ± 2.7	48.2 ± 5.1	0.001	<0.0001	25.8 ± 2.9	13.8 ± 2.3	0.001	0.03

γ_Na_ was calculated from chord conductance, γ_Ca_ was calculated from slope conductance.

Data are shown as mean ± SD, p values were estimated with Student’s paired t-test relative to no-PAM control.

Therefore, when examined at the single-channel level, with supersaturating agonist concentrations, and nominal absence of inhibitors and blockers, GNE-9278 produced negligible effects on GluN2A receptor conductance and gating, whereas for GluN2D receptors, it increased substantially both the unitary current amplitude and the channel Po, by increasing the open durations and reducing the closed durations [27].

### GNE-9278 stabilized NMDA receptors in open states

**Fig. 3:**
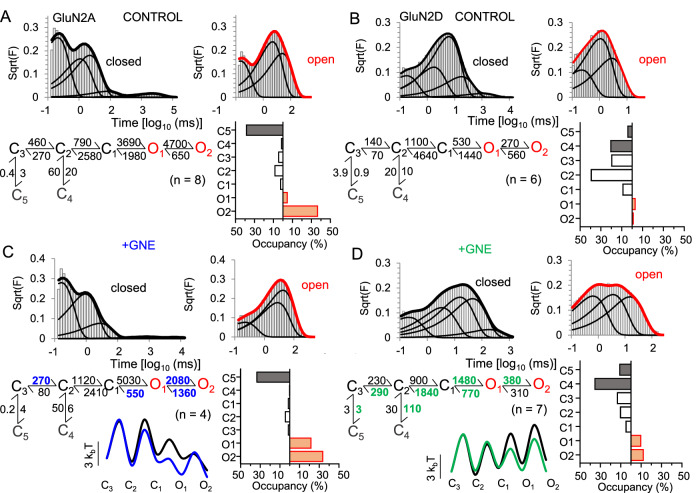
GNE-9278 stabilizes GluN1/GluN2A and GluN1/GluN2D receptors in open states. Histograms illustrate closed and open dwell times detected in one recording for GluN1/GluN2A (**A**) and for GluN1/GluN2D (**B**), without and with GNE-9278 (50 μM) (**C**–**D**). Superimposed, are lines representing the probability density functions (thick) and time components (thin) calculated with the best fitting state model (5C3O). The corresponding canonical state models (5C2O, illustrated) are illustrated below, with rates for each transition (s^-1^) displayed as averages (rounded to the 10^th^ percentile) of the values obtained by fitting the model to the data in each recording. Statistical significance of differences observed with GNE-9278 relative to control were determined with a Student’s t test, and rates with *p* <  0.05 are highlighted (bold, color). Also illustrated are energy landscapes (lines) and state occupancies (bar graphs) calculated from the respective models.

To evaluate the impact of GNE-9278 on the NMDA receptor gating mechanism, we modeled the recorded single-channel activity with procedures described previously [16]. Briefly, we fitted models that had increasing numbers of states to the observed sequence of closed and open dwell times in each recording. For each model, we used the calculated probability density function to determine its log likelihood function and selected the model with the minimum number of states necessary for the best fit. We found that in most of our recordings, models with five closed and three open states (5C3O) produced the best fits to the data as illustrated in (Figs. 3A and 3B). In each data set, one or two recordings revealed a more complex gating pattern, which required models with five closed and four open states (5C4O) for best fit, indicative of modal gating [27, 28]. Based on these observations, we conclude that GNE-9278 does not change the receptors’ reaction mechanism but only the kinetics with which receptors cycle among the various states.

To estimate transition rates and to determine whether GNE-9278 affects preferentially specific steps in the gating reaction, we chose to use the canonical 5C2O reaction mechanism [19]. This approach ignores modal gating, although it is present in all the recordings, and therefore provides only a general estimate of changes in kinetics. However, by modeling all recordings with the same mechanism and considering each recording in its entirety, rather than separating activity by mode, we can obtain meaningful statistics for the 12 transitions considered explicitly in this model, while also retaining mechanistic information for any observed changes.

For GluN2A receptors, GNE-9278 had the most pronounced effect on the closing rate C_1_ ← O_1_, which was ~4-fold slower when the drug was present. The energy landscape calculated from this reaction mechanism indicated that GNE-9278 stabilized receptors in open states and increased the occupancy of open states at equilibrium (Fig. 3C), as we reported previously for a truncated GluN2A receptor [11]. For GluN2D receptors, we observed a more complex pattern of kinetic changes, with the most notable differences occurring for transitions into and out of state C_1_. The free-energy fluctuations calculated with these rates indicated that GNE-9278 reduced the energy barriers for entry into open states and stabilized open states. Overall, these changes resulted in substantial increase in the occupancy of open states at equilibrium (Fig. 3D).

The structural arrangement represented by the transitions postulated by this kinetic model remain under intense investigation. We proposed recently that the C_1_ ↔ O_1_ transition, which represents the last step necessary for ionic permeation, represents the bending of the GluN1-M3 helices [11]. Our finding that GNE-9278, which binds to the extracellular side of the GluN1 transmembrane domain, stabilized open-pore conformations supports this interpretation.

### GNE-9268 increased the Ca^2+^ permeability of GluN2D receptors

**Fig. 4:**
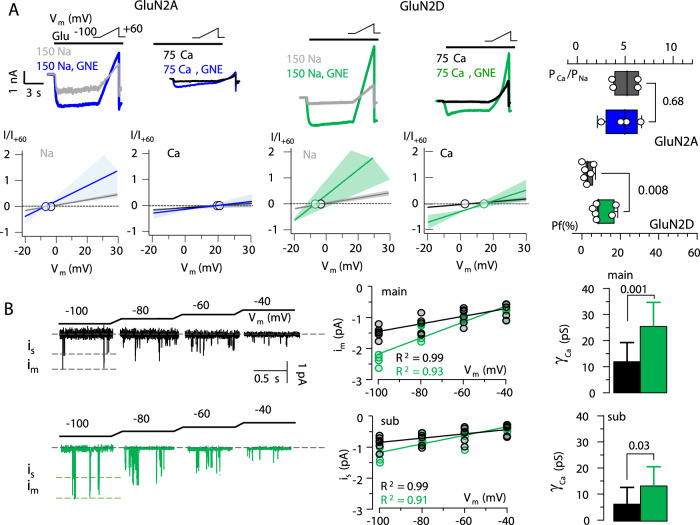
GNE-9278 increases the Ca^2+^ permeability and conductance of GluN1/GluN2D receptors. **A** Representative whole-cell current traces recorded from GluN2A (*left*) and GluN2D (*right*) receptors in response to Glu (1 mM) at the indicated voltages (V_m_), in Na^+^ (*left*, 150 mM, gray) and Ca^2+^ (right, 75 mM, black), without or with GNE-9278 (50 µM, color). Linear fits (means ± 95% CI) to currents recorded between –20 and +20 mV, and the measured reversal potentials (E_rev_, circles) without (black) and with GNE-9278 (color). Relative Ca^2+^ permeability (P_Ca_/P_Na_) calculated from Ca^2+^-dependent changes in E_rev_ (ΔE_rev_) and the corresponding fractional Ca^2+^ current (Pf) calculated by Eq. 3. **B** Left, representative unitary current traces recorded from cell-attached patches with Ca^2+^ (75 mM) as the only permeant ion, without (black) or with (color) GNE-9278, at four membrane potentials. Dotted lines mark levels for zero, sub and main current levels. Middle, summary of measured i/V relationships (n > 10, for each), with superimposed linear fits, and bar graph (*right*) with calculated unitary Ca^2+^ conductance (γ_Ca_). Significance determined with the Student’s t-test.

Given the strong effect of GNE-9278 on the GluN2D receptor unitary sodium conductance (Fig. 2B) we next examined its effect on this receptor’s Ca^2+^ permeability by measuring Ca^2+^-dependent changes in reversal potential (E_rev_) in the absence and presence of GNE-9278, using the biionic ion method [2]. We recorded whole-cell currents while ramping the membrane potential, in low (0.5 mM) and high (75 mM) Ca^2+^ concentrations. For GluN2A receptors, we measured reversal potential shifts (ΔE_rev_) of 23.5 ± 4.4 mV, corresponding to a relative Ca^2+^ permeability (P_Ca/_P_Na_) of 4.2 ± 1.3, as reported previously [4]. In the presence of GNE-9278, we measured ΔE_rev_, 25.1 ± 4.1 mV, corresponding to P_Ca_/P_Na_, 4.1 ± 1.2 indicating that at pH 8.0 GNE-9278 had no effect on the Ca^2+^ permeability of GluN2A receptors, as reported previously (Fig. 4A) [29].

For GluN2D receptors, the Ca^2+^-induced shift in reversal potential (ΔE_rev_) was 7.0 ± 4.9 mV, corresponding to a relative Ca^2+^ permeability (P_Ca/_P_Na_) of 1.4 ± 0.5, lower than previously reported [30]. Given our use of high (75 mM) Ca^2+^ concentrations, this lower value may reflect Ca^2+^-dependent decreases in P_Ca_/P_Na_ calculated by the bionic method at extreme Ca^2+^ concentrations, as has been previously reported **(**Fig. S2, Table S2) [22, 23]. In the presence of GNE-9278, we measured larger ΔE_rev_, 19.6 ± 5.0 mV, corresponding to P_Ca_/P_Na_, 2.9 ± 1.0, which represents a 2-fold increase over control values (Fig. 4A). This result indicates that in addition to increasing the open probability and the unitary sodium conductance of GluN2D receptors, GNE-9278 also increased their relative Ca^2+^ permeability, which suggests an increase in the unitary Ca^2+^ conductance.

To measure directly the GluN2D receptor unitary Ca^2+^ conductance, we recorded single-channel currents from cell-attached patches with Ca^2+^ as the only permanent ion (75 mM) at four holding potentials (-100, -80, -60 and -40 mV). At -100 mV. We observed two open levels with amplitudes of 0.8 ± 0.1 pA, and 1.4 ± 0.1 pA, corresponding to unitary conductances of 7 ± 2 pS and 12 ± 2 pS. In the presence of GNE-9278, these values increased 2-fold to 14 ± 2 pS and 26 ± 3 pS, respectively (Fig. 4B). The much larger increase in the unitary conductance of Ca^2+^ than of Na^+^ (100% vs. 30%), explains the GNE-9278-dependent increase in relative Ca^2+^ permeability.

## Discussion

We examined the effect of GNE-9278, a pan-NMDA receptor positive allosteric modulator on the biophysical properties of GluN1/GluN2A and GluN1/GluN2D receptors. We found that GNE-9278 had similar potencies ( ~ 10 µM) on both receptor types, but had higher efficacies for GluN2D than GluN2A receptors, consistent with previous reports [10]. We performed our study in low proton concentrations (pH 8), to prevent tonic inhibition by protons and focus on intrinsic effects of GNE-9278. We found much lower levels of potentiation by GNE-9278 than in studies done in physiological conditions (pH 7.3). This result indicates that in addition to an intrinsic effect on channel kinetics, GNE-9278 also prevents tonic inhibition by protons.

In the absence of proton inhibition, GNE-9278 produced only a small increase in GluN2A currents. This increase resulted from longer openings due to slower rate for the closing step C_1_ ← O_1_, with no changes in the unitary sodium amplitude, or Ca^2+^ permeability. In contrast, GNE-9278 increased substantially the GluN2D receptor open probability (4-fold), sodium conductance (1.3-fold), Ca^2+^ conductance (4-fold), and Ca^2+^ permeability (2-fold). However, despite the large increase in GluN2D current produced by GNE-9278, the resulting activity (Po, 0.3) remained well below that of GluN2A receptors (Po, 0.5). Similarly, when considering the effects of GNE-9278 on sodium conductance and Ca^2+^ permeability, although GNE-9278 increased both these parameters substantially for GluN2D, they remained lower than the corresponding values for GluN2A. One possibility is that GNE-9278 binding to the GluN1 subunit stabilizes open states of both GluN2A and GluN2D receptors in a way that compensates for unfavorable interactions present in GluN2D but not in GluN2A. This interpretation is supported by the observation that much of the potentiation observed in physiological pH represents relief from tonic proton inhibition. Similarly, because GNE-9278 binds close to the channel gate and only interacts with GluN1 residues it likely acts downstream from the structures that confer low Po onto GluN2D receptors and serves to overpower these deficits. This interpretation is consistent with the observation that GNE-9278 renders NMDA receptors less sensitive to extracellular allosteric modulators [10, 25].

Our finding that GNE-9278 increased the GluN2D receptor conductance and Ca^2+^ permeability was unexpected. Allosteric modulators are state-dependent ligands that bind to sites other than the orthosteric agonist-binding site and act by changing receptor kinetics not channel properties. Recently, an exception to this rule was reported for EU1622-240, which increased the Po of GluN2B receptors but reduced their unitary conductance [31]. Our observation that GNE-9278 alters conductance and permeability of NMDA receptors in a subunit-dependent fashion merits further investigation. Notably, in cortical neurons, GNE-9278 increased the level of Ca^2+^-dependent desensitization of NMDA receptor currents [32]. This observation may reflect increased Ca^2+^ flux in the presence of GNE-9278, with increased calmodulin-mediated auto-inhibition [9, 33–35].

These results add to the growing body of literature describing the mechanistic action of positive allosteric modulators. Together they support existing observations that GNE-9278 modulates NMDA receptors to increase frequency and duration of channel opening and demonstrate subunit-dependent effects on unitary conductance and relative Ca^2+^ permeability. To date, this panel of effects on NMDA receptor currents remains unique to GNE-9278.

## Supplementary information

Table S1, Table S2, Figure S1, Figure S2

## Data Availability

All the data is represented in the article.
