# Supplementary material for: The positive allosteric modulator GNE-9278 increases gating, conductance, and Ca2+ permeability for GluN2D-containing NMDA receptors
Source: Neuropsychopharmacology. 2026 Jan 7;51(8):1395–401. doi: 10.1038/s41386-025-02308-8 (PMC13291320; doi:10.1038/s41386-025-02308-8)
Supplement: Supplementary file 1 — Table S1, Table S2, Figure S1, Figure S2 [file 41386_2025_2308_MOESM1_ESM.pdf]

Table S1. Microscopic kinetics

Receptor	GNE μM	I (pA)	P_o	MOT (ms)	MCT (ms)	n	Events total	Recording (min)
GluN2A	0	9.4 ± 2.7	0.47 ± 0.14	5.3 ± 2.3	5.3 ± 2.0	8	1,274,460	227
	50	8.5 ± 1.7	0.59 ± 0.04	4.8 ± 0.7	3.5 ± 1.5	4	499,367	62
GluN2D	0	5.2 ± 0.7<br>6.3 ± 0.4	0.06 ± 0.05	1.1 ± 0.5	24.0 ± 9.4	6	288,649	131
	50	7.0 ± 0.7<br>8.2 ± 0.4	0.24 ± 0.07*	3.2 ± 1.0*	10.8 ± 4.8	7	479,004	104

*Data are shown as mean ± SD. *Student's paired t-test relative to N1/N2D without GNE*

Table S2: Relative Ca^{2+} permeability calculations by two methods.

Receptor Method	GNE (µM)	Ca^{2+} (mM)	E_{rev} (mV)	ΔE_{rev} (mV)	P_{Ca}/P_{Na}	Pf(%)	n	P-value*
GluN2A Biionic	0	0.2	-3.3 ± 2.6				4	
		75	20.3 ± 5.3	23.6 ± 4.4	4.2 ± 1.3	21.4 ± 6.4	4	
	50	0.2	-8.5 ± 2.4				4	
		75	16.6 ± 5.9	25.1 ± 4.1	4.1 ± 1.2	20.6 ± 6.1	4	0.68
GluN2D Biionic	0	0.5	-5.3 ± 5.1				6	
		75	1.7 ± 7.1	7.0 ± 4.9	1.4 ± 0.5	7.3 ± 2.3	6	
	50	0.5	-7.9 ± 5.1				6	
		75	11.6 ± 6.6	19.5 ± 5.0	2.9 ± 1.0	15.4 ± 5.5	6	0.008
GluN2D Monovalent	0	0.2	-4.8 ± 5.7				7	
		2.0	-3.2 ± 5.7	1.5 ± 1.3	2.3 ± 2.0	11.6 ± 10.2	7	
	50	0.2	-4.8 ± 5.7				7	
		2.0	-1.5 ± 6.2	3.3 ± 1.5	5.2 ± 2.9	26.5 ± 14.8	7	0.04

*Data are shown as mean ± SD. *Student's paired t-test relative to N1/N2D without GNE*

Figure S1: GNE-9278 reversibly potentiates NMDA receptor whole-cell currents.

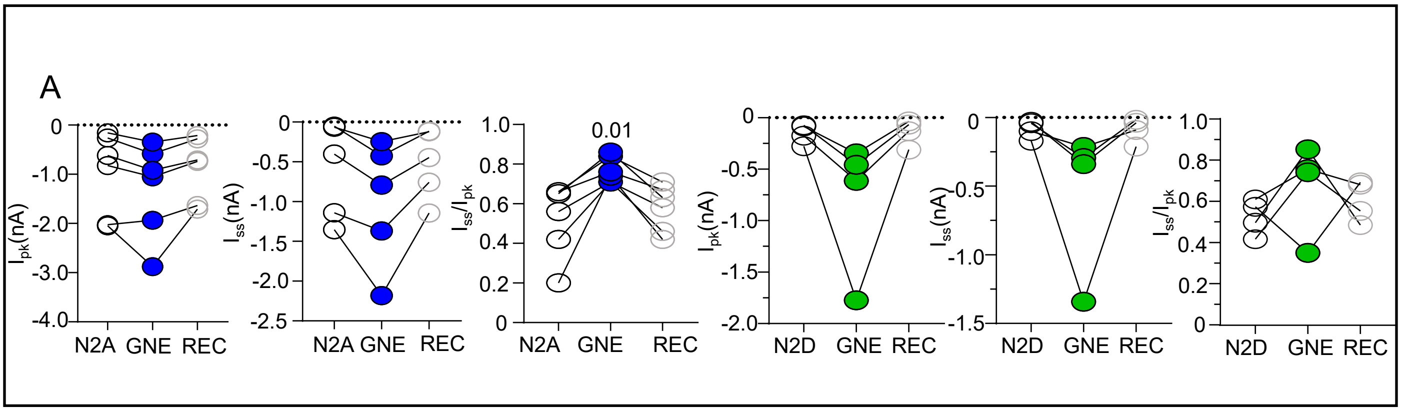

Figure S1: GNE-9278 reversibly potentiates NMDA receptor whole-cell currents. A. Summary of measured current amplitudes at peak (Ipk) and at steady-state (Iss), and on calculated current desensitization (Iss/Ipeak) for N1/N2A (blue) and N1/N2D (green) receptors.

Figure S2: GNE-9278 increases the Ca^{2+} permeability of GluN2D receptors measured by the high monovalent method.

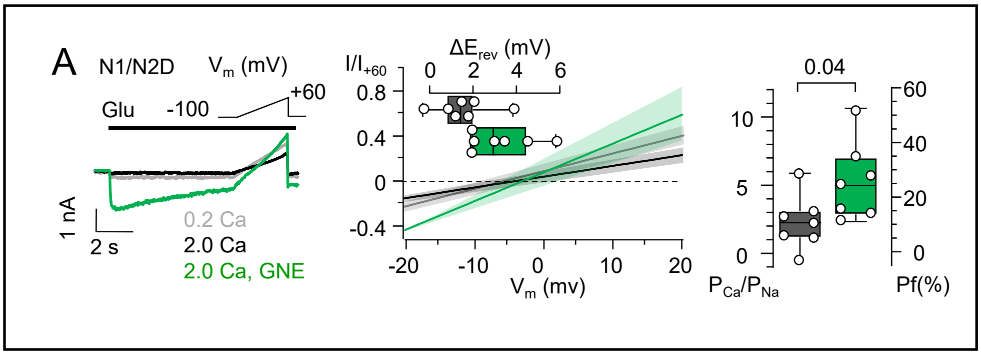

Figure S2: GNE-9278 increases the Ca^{2+} permeability of GluN2D receptors. (A) *Left*, representative whole-cell current traces recorded from GluN2D receptors in response to Glu (1 mM) at the indicated voltages (V_m), in low (0.2 mM, gray) and high Ca^{2+} (2 mM, black), and with high Ca^{2+} and GNE-9278 (green). *Middle*, linear fits (means ± 95% CI) to currents recorded between –20 and +20 mV, and the measured reversal potentials (ΔE_{rev}) without (black) and with GNE-9278 (green) (*insert*). *Right*, relative Ca^{2+} permeability (P_{Ca}/P_{Na}) estimated from $\Delta e_{rev,}$ and the corresponding fractional Ca^{2+} current (Pf) were calculated as described previously [2]. Significance determined with the Student's t-test.
